# Pathology of Fibrosis in Crohn's Disease—Contribution to Understanding Its Pathogenesis

**DOI:** 10.3389/fmed.2020.00167

**Published:** 2020-05-05

**Authors:** Nina Zidar, Cord Langner, Miha Jerala, Emanuela Boštjančič, David Drobne, Aleš Tomažič

**Affiliations:** ^1^Faculty of Medicine, Institute of Pathology, University of Ljubljana, Ljubljana, Slovenia; ^2^Diagnostic and Research Institute of Pathology, Medical University of Graz, Graz, Austria; ^3^Department of Gastroenterology, University Medical Centre Ljubljana, Ljubljana, Slovenia; ^4^Department of Abdominal Surgery, University Medical Center, Ljubljana, Slovenia

**Keywords:** Crohn's disease, fibrosis, stenosis, pathology, pathogenesis

## Abstract

**Background:** Despite significant progress in the research of fibrosis in various organs, fibrosis remains a poorly understood complication of Crohn's disease (CD). We analyzed pathologic features of fibrosis and inflammation in CD and compared them with the normal bowel, aiming to clarify whether fibrosis in CD pathogenetically resembles fibrosis in other organs.

**Methods:** Resection specimens from 30 patients with CD were included. Normal bowel from resection specimens of colorectal carcinoma was used for comparison. Trichrome Masson staining, immunohistochemistry for α-smooth muscle actin, fibroblast activation protein, CD34 and erg, *in situ* hybridization for TGF-β1 and analysis of selected fibrosis-related microRNAs were performed.

**Results:** In normal bowel, CD34-positive fibroblasts/pericytes were detected in the submucosa and subserosa, particularly around blood vessels. In CD, fibrosis prevailed in the submucosa and subserosa, together with proliferation of myofibroblasts and disappearance of CD34-positive fibroblasts/pericytes. TGF-β1 was present in the lamina propria in normal bowel and CD, and in deeper parts of the bowel wall in CD. MicroRNAs *miR-29c, miR-155 miR-150*, and *miR-155*, which have been demonstrated to contribute to fibrosis in various organs, showed significant deregulation in CD.

**Conclusions:** Distribution of fibroblasts/pericytes in the submucosa and subserosa of normal bowel, their disappearance in fibrosis in CD, together with the appearance of myofibroblasts, suggest that fibroblasts/pericytes are the most likely source of myofibroblasts in CD. Furthemore, fibrosis-related microRNAs showed deregulation in fibrotic areas. Pathogenesis of fibrosis in CD is thus comparable to fibrosis in other organs, in which myofibroblasts are the key effector cells, and pericytes have emerged as the main origin of myofibroblasts. Fibrosis in CD should be regarded as a result of (over)response of the bowel wall to the presence of inflammation in deep structures of the bowel wall, presenting another example of a common pathogenetic pathway of fibrosis development.

## Introduction

Extensive research in recent decades has provided new insights into the pathogenesis of inflammatory bowel diseases, including Crohn's disease (CD) and has enabled the development of new treatment modalities. Despite significant progress, fibrosis remains a poorly understood complication of CD, in terms of pathogenesis, diagnosis and treatment (1–4). The introduction of new immunosuppressive drugs, such as anti-TNF antibodies two decades ago, seemed promising and it was expected to reduce the incidence and severity of fibrosis. However, subsequent studies have shown that, despite early treatment, the incidence of stenosis and the need for surgery have not significantly changed over the last 25 years (5–7). Fibrosis with subsequent stricture formation develops in up to 70 % of patients with CD within 10–20 years after diagnosis (8). Moreover, stricture recurs at anastomotic sites in up to 50% of patients, necessitating multiple surgeries, leading to significant morbidity (9, 10).

Fibrosis is the final common pathway in organ failure in diseases of various organs, including the liver, kidney, lung and heart (11–13). It is believed to result from tissue damage due to chronic injury and/or inflammation and impaired mechanisms of wound healing, leading to progressive scarring and organ damage (11, 12, 14). Myofibroblasts are considered to be the key effector cell in fibrosis, being responsible for the synthesis of the extracellular matrix proteins (13, 15, 16). Fibrosis has been generally regarded as being permanent and irreversible, and very few therapies for fibrosis are available, mostly of limited efficacy. However, there is emerging evidence suggesting that fibrosis can be reversed in some diseases, for example in the liver, if the causative agent is removed (17–20). It remains to be determined whether the reversibility of fibrosis in CD is a fact or fiction, as stated by Bettenworth and Rieder (10).

In contrast to recent progress in our understanding of fibrosis development in the liver, kidney and heart, there has been little progress in our understanding of fibrosis in the bowel, including CD. In particular, pathologic studies of fibrosis in CD are relatively rare (21). We believe that analyzing the pathologic features of fibrosis in CD might contribute to our understanding of its pathogenesis (22). Using simple, widely used methods in pathology, we analyzed the distribution and extent of fibrosis, fibroblasts and myofibroblasts in CD in comparison to normal bowel wall. We also analyzed the expression of some microRNAs, which have been demonstrated to contribute to fibrosis in various organs.

## Methods

### Patients

The study was approved by the National Medical Ethics Committee, Ministry of Health, Republic of Slovenia (No 0120-139/2019/4). It included 30 patients with CD who had undergone bowel resection. The control group consisted of normal bowel (colon and ileum) from 10 patients who had undergone resection for colorectal carcinoma. Diagnosis of CD was made on the basis of clinical, radiological, endoscopic and histologic findings (23, 24). For the purpose of this study, the most important demographic and clinical data were collected (CD type, duration and maximal extent of the disease, therapy, indication for surgery).

Resection specimens that had been fixed in 10% buffered formaldehyde were handled according to standard procedures. Samples were taken from the most stenotic regions, from inflamed mucosa and ulcers as well as from macroscopically normal mucosa. All samples were embedded in paraffin and cut at 4 μm, stained with haematoxylin and eosin and analyzed according to standard criteria (25).

All slides were reviewed for the purpose of this study. Two slides with corresponding paraffin blocks were chosen from each patient for further analysis: one with the most severe fibrosis and one with the most severe inflammation (without fibrosis) on histologic examination. Samples with fistula and samples from anastomosis were not included.

### Trichrome Masson Staining and Immunohistochemistry

All slides were stained with Masson's trichrome with aniline blue (04-01802, Bio-Optica, Milan, Italy) for detection of fibrosis. With this stain, cytoplasm stains red, nuclei black and collagen fibrous tissue blue.

For immunohistochemistry, we used antibodies against α-smooth muscle actin (α-SMA) (MA1-26017, Labvision, UK, dilution 1:20), fibroblast activation protein (FAP, EPR20021, Abcam, UK, dilution 1:100) and for double immunostaining, antibodies against CD34 (MA5-15331, Labvision, UK, dilution 1:20) and erg (701183, Labvision, UK, dilution 1:20). Sections were treated with biotinylated secondary antibody, followed by incubation with peroxidase conjugated streptavidin (iVIEW™ DAB Detection Kit, Ventana Medical System, Tucson, AZ, USA). Visualization of the immunoreaction was carried out with 3.3' diaminobenzidine. Finally, sections were counterstained with hematoxylin.

Normal structures of the bowel wall served as a positive control. α-SMA positivity was found in pericryptal myofibroblasts, muscularis mucosae, muscularis propria and smooth muscles in blood vessel walls. There was no staining of the normal bowel wall for FAP. Double immunostaining exhibited a red cytoplasmic reaction for CD34 and brown nuclear reaction for erg. Both brown and red stainings were found in endothelial cells, whereas red cytoplasmic staining was found in fibroblasts and pericytes. Negative controls omitting the primary antibodies were included in every run of samples.

### *In situ* Hybridization and microRNA Expression Analysis

For *in situ* hybridization for TGF-β1, mRNA transcripts were detected using a commercially available *in situ* hybridization RNAscope 2.0 kit (Cat No. 400881, Advanced Cell Diagnostics, Hayward, CA, USA) following the manufacturer's instruction. Briefly, tissue sections were first incubated for 1 h at 60°C, followed by deparaffinization, incubation with Pretreatment Reagent 1 for 10 min at room temperature, Pretreatment Reagent 2 for 15 min at 100°C and Pretreatment Reagent 3 for 30 min at 40°C. Tissue sections were then incubated with probe for 2 h at 40°C. Diaminobenzidine was used as chromogen. Sections were counterstained with hematoxylin. Positive reaction appeared as dot-like signals in the cytoplasm and nuclei.

For microRNA analysis, RNA was isolated and quantified from formalin-fixed paraffin-embedded tissue sections as previously described (26). Twelve samples from fibrotic areas of CD, 7 samples from inflamed areas of CD and 14 samples of normal colon were included. Expression of 3 miRNAs, associated with different pathways of fibrosis (24), i.e*., miR-29c, miR-150*, and *miR-155* was analyzed relatively to *miR-16, miR-103a-3p, miR-191*, and *let-7a* using miRCURY LNA miRNA assay and detection system (Qiagen) according to the manufacturer instruction.

## Results

The most important demographic and clinical data at the time of surgery are presented in Table 1. The cohort included 14 men and 16 women, aged 25 to 58 years (39.8 ± 9.4). The duration of the disease prior to surgery ranged from zero to 35 years (10.3 ± 9.9). The most frequent indication for resection was stenosis. Twenty-two patients were on immunosuppressive medications.

**Table 1:** Demographic and clinical data of patients with Crohn's disease.

**Male: female**	**14: 16**
Age (years) (mean ± SD)	39.8 ± 9.4
Duration of disease (years) (mean ± SD)	10.3 ± 9.9
**Indication for surgery**	
inflammation, abscess, fistulas	7 (23.3%)
perforation	2 (6.7%)
ileus	3 (10%)
stenosis	18 (60%)
**Extent of disease**	
ileum	4 (13.3%)
colon	6 (20%)
ileum and colon	20 (66.7%)
**Immunosuppressive medication**	
methotrexate	4 (13.3%)
infliximab	6 (20%)
azathioprine	14 (46.7%)
mycophenolate mofetil	2 (6.7%)
adalimumab	11 (36.7%)

Histological examination showed features of CD in varying proportions: erosions and ulcers, architectural abnormalities, chronic and active inflammation, granulomas, transmural lymphoid aggregates, fibrosis, and neural hyperplasia. The muscularis mucosae was often distorted or rarely duplicated, particularly in healing ulcers, and muscularis propria was occasionally hypertrophic. The intensity of inflammation in fibrotic regions varied, but inflammation was present in all cases. No significant differences were observed between the ileum and colon samples regarding features related to fibrosis.

### Masson's Trichrome Staining

**Figure 1:**
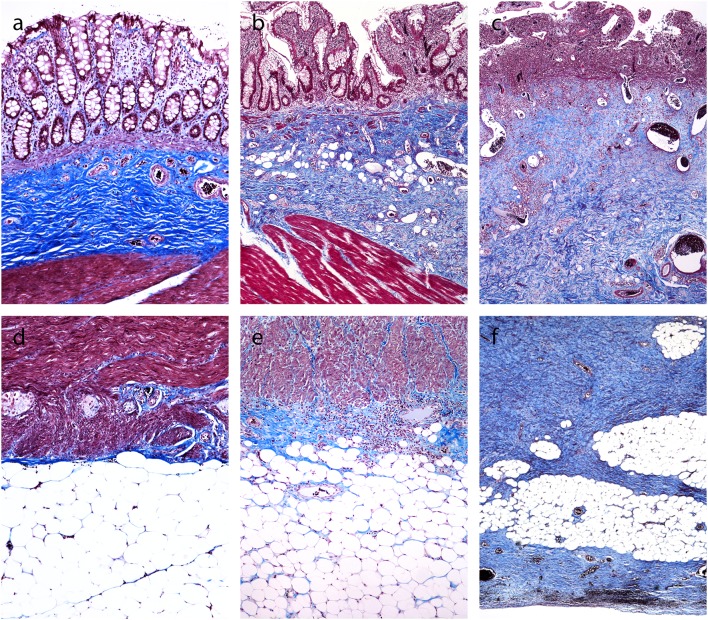
Trichrome Masson staining of the normal colon and Crohn's disease with and without stenosis (inner part of the bowell wall in A, B, C, outer part of the bowel wall in D, E, F). **(a,d)**. Normal colon: blue-colored fibrous tissue in the submucosa (A), but not in the lamina propria and subserosa **(d)**. **(b,e)** Crohn's disease without stenosis: blue-colored fibrous tissue in the submucosa **(b)**, but not in the lamina propria **(b)**, and very little staining in the subserosa **(e)**. **(c,f)** Crohn's disease with stenosis: abundant blue-colored fibrous tissue in the submucosa **(c)** and subserosa **(f)**.

In normal colon, the blue staining of collagenous fibrous tissue was present mainly in the submucosa (Figure 1A). Some narrow bands of blue staining were seen between the smooth muscle fibers of the muscularis propria and very little or none in the subserosa (Figure 1D) and beneath the mesothelial cells.

In CD, collagenous fibrous tissue was increased in the submucosa (Figures 1B,C). There were two patterns in the subserosa. In inflamed areas (without significant stenosis), there was either no fibrosis or there were small areas of fibrosis close to the outer part of the muscularis propria (Figure 1E). In CD with stenosis, there was abundant fibrosis in subserosal tissue (Figure 1F). It was dense, unevenly distributed, either homogenous or in irregular bands, focally extending to the mesothelial layer on the serosal surface.

The blue staining of collagen fibrous tissue was mostly not present in the lamina propria, in either normal mucosa or CD (Figures 1A–C). The only exception was in the deep lamina propria in patients with CD with healed ulcers, accompanied either by destruction or duplication of the muscularis mucosae. In the latter cases, the blue staining of collagenous fibrous tissue was found in the deeper part of the lamina propria and/or between the duplicated layers of the muscularis mucosae.

### Immunohistochemistry

#### α-Smooth Muscle Actin

In normal colon, a positive reaction for α-SMA was found in pericryptal myofibroblasts, in smooth muscle cells of the muscularis mucosae and muscularis propria, and in blood vessel walls (not shown).

**Figure 2:**
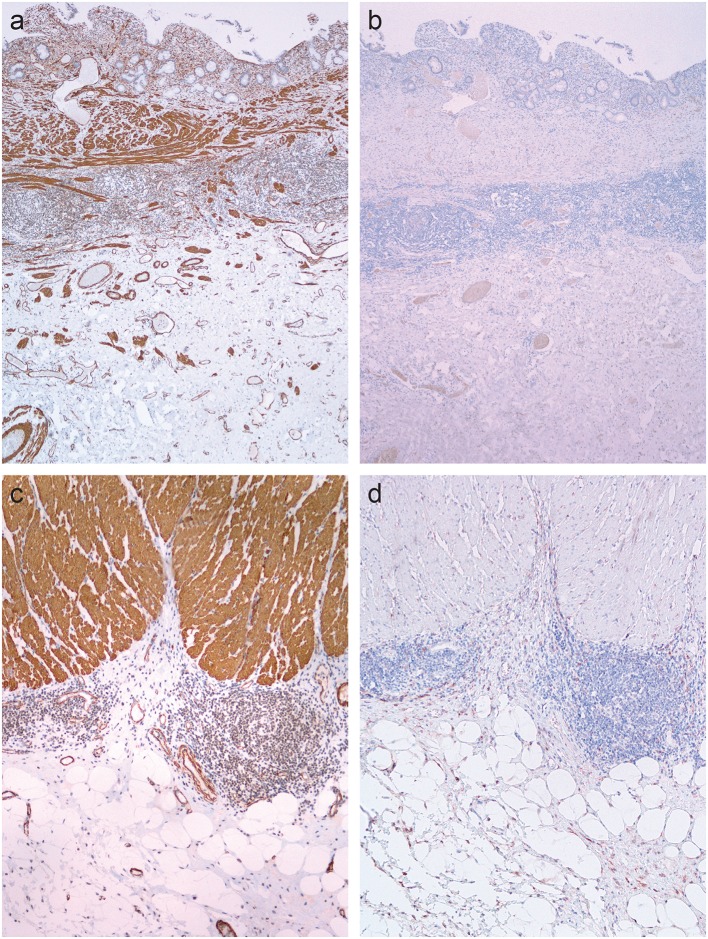
Inflamed areas in Crohn's disease. **(a,c)** Immunohistochemistry for α-smooth muscle actin shows positive reaction in the smooth muscles of the muscularis mucosae, muscularis propria and blood vessel walls. **(b,d)** Immunohistochemistry for fibroblast activation protein does not show any positive reaction.

**Figure 3:**
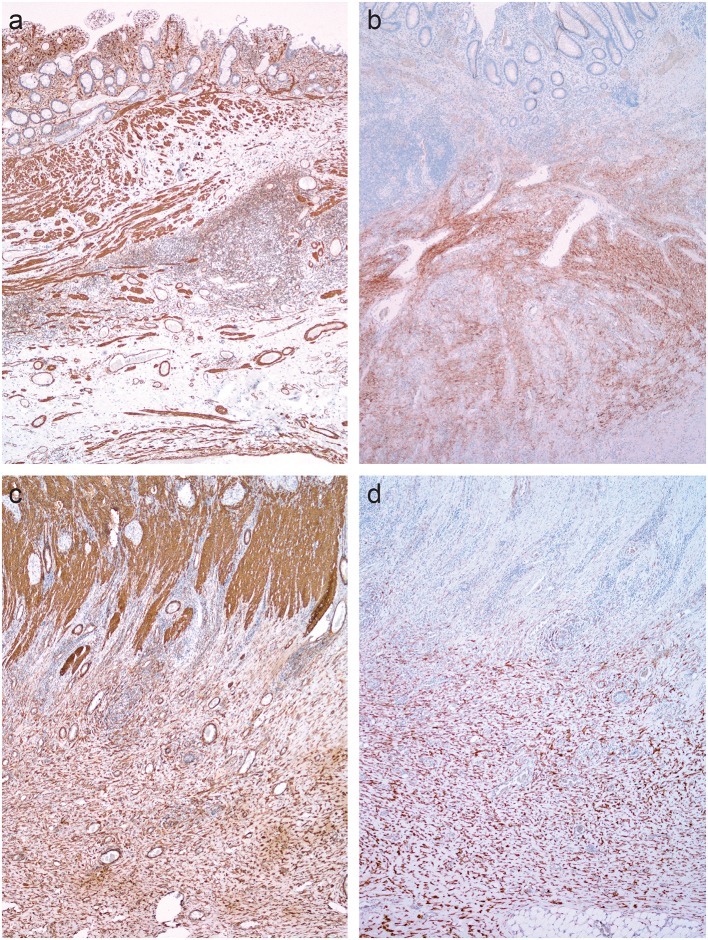
Fibrostenotic areas in Crohn's disease. **(a,c)** Immunohistochemistry for α-smooth muscle actin shows positive reaction in the smooth muscles of the muscularis mucosae, muscularis propria and blood vessel walls, and in myofibroblasts in the submucosa and in the subserosa. **(b,d)** Immunohistochemistry for fibroblast activation protein shows positive reaction in myofibroblasts in the submucosa and subserosa, but not in smooth muscles of the muscularis mucosae, muscularis propria and blood vessel walls.

In CD, α-SMA-positive myofibroblasts were identified as spindle-shaped cells with strong cytoplasmic positivity. In CD with inflammation, they were very few in the submucosa (Figure 2A) and few or none in the subserosa (Figure 2C). In CD with fibrostenosis, they were present in the submucosa (Figure 3A), and they were abundant in areas of dense subserosal fibrosis (Figure 3C).

In some cases of CD, the muscularis mucosae disappeared or was markedly distorted, admixed with inflammatory cells and fibrosis (Figure 3A), making it difficult to distinguish smooth muscle cells of the distorted muscularis mucosae from myofibroblasts.

### Fibroblast Activation Factor

In normal colon, there was no staining for FAP (not shown). In CD, there was no staining for FAP in areas of inflammation without fibrosis (Figures 2B,D). FAP staining was found in myofibroblasts in the submucosa (Figure 3B) and subserosa (Figure 3D) in samples from fibrostenotic areas of CD. There was no staining of the smooth muscles in muscularis mucosae and muscularis propria (Figures 2, 3B,D).

### CD34+erg

**Figure 4:**
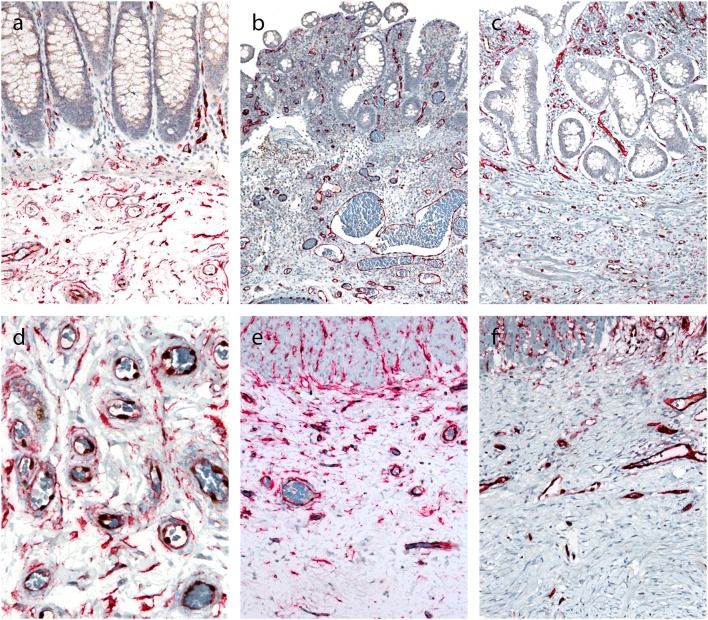
Double immunohistochemical reaction for CD34 and erg in the normal colon and Crohn's disease with and without stenosis. **(a,d)** Normal colon: double immunostaining for CD34 (red) and erg (brown) in endothelial cells in blood vessels, and red cytoplasmic reaction in spindle-shaped cells in the submucosa **(a)** and subserosa, particularly around blood vessels **(d)**. **(b,e)** Crohn's disease without stenosis: double immunostaining for CD34 (red) an erg (brown) in endothelial cells in blood vessels in the lamina propria and submucosa; no spindle-shaped cells with red cytoplasmic reaction **(b)**. Double immunostaining for CD34 (red) and erg (brown) in endothelial cells in blood vessels, and red cytoplasmic reaction in spindle-shaped cells in the subserosa, particularly around blood vessels **(e)**. **(c,f)** Crohn's disease with stenosis: double immunostaining for CD34 (red) an erg (brown) in endothelial cells in blood vessels in the lamina propria and submucosa **(C)** and in fibrosis in subserosa **(f)**; no spindle-shaped cells with red cytoplasmic reaction.

In normal colon, double immunostaining for CD34 and erg was found in endothelial cells, while a red cytoplasmic reaction was observed in spindle-shaped cells, which were abundant in the submucosa (Figure 4A) and in the subserosa, particularly around blood vessels (Figure 4D) and beneath mesothelial cells.

In CD, double immunostaining for CD34 and erg was found in endothelial cells in the submucosa (Figures 4B,C) and in the subserosa (Figures 4E,F). CD34-positive spindle-shaped cells were present in the subserosa in CD without stenosis (Figure 4E), but disappeared in the subserosa in CD with stenosis (Figure 4F).

### mRNA *In situ* Hybridization for Transforming Growth Factor-β1 (TGF-β1)

**Figure 5:**
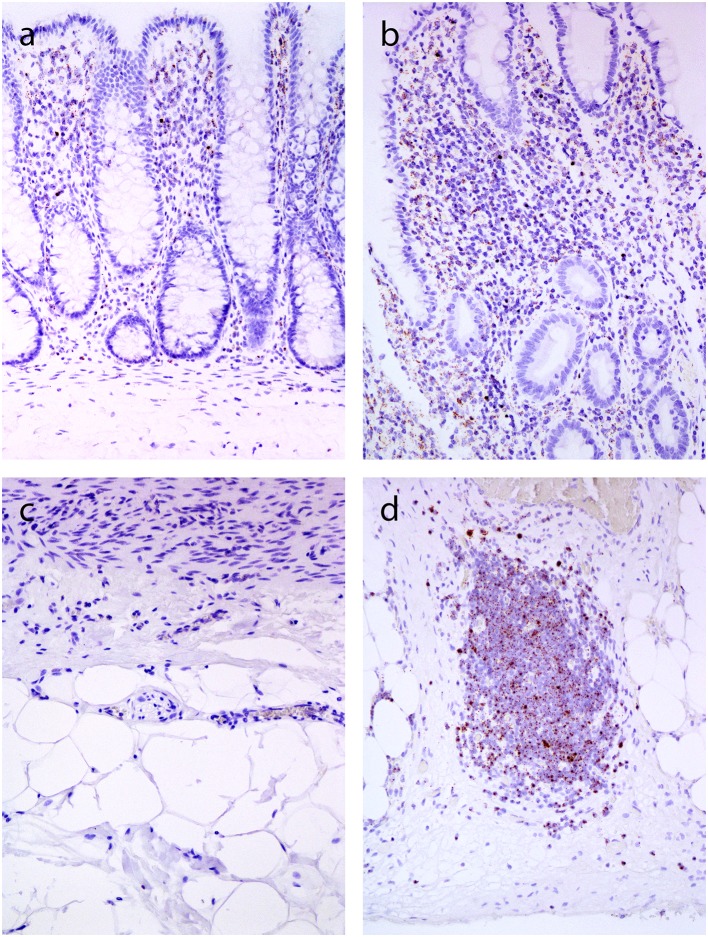
*In situ* hybridization for TGF-β1 (inner part of the bowell wall in A and B, outer part of the bowel wall in C and D). and **(c)**. Normal colon mucosa: positive reaction in lymphocytes in the lamina propria **(a)**, no positivity in the subserosa **(c)**. **(b,d)** Crohn's disease: positive reaction in lymphocytes in the lamina propria **(b)**, and in lymphoid aggregate in the subserosa **(d)**.

In normal bowel, positive reaction with dot-like signals was present in lymphocytes and lymphatic follicles in the lamina propria and submucosa (Figure 5A), while no staining was observed in deeper parts of the bowel wall (Figure 5C). In CD, a positive reaction was also found in lymphocytes and lymphoid follicles in the lamina propria and submucosa (Figure 5B). In both normal bowel and CD, the intensity of staining correlated with the density of lymphoid infiltration. In CD, positive signals for TGF-β1 were found in lymphoid aggregates throughout the bowel wall, including subserosal fibrosis (Figure 5D).

### microRNA Expression Analysis

**Figure 6:**
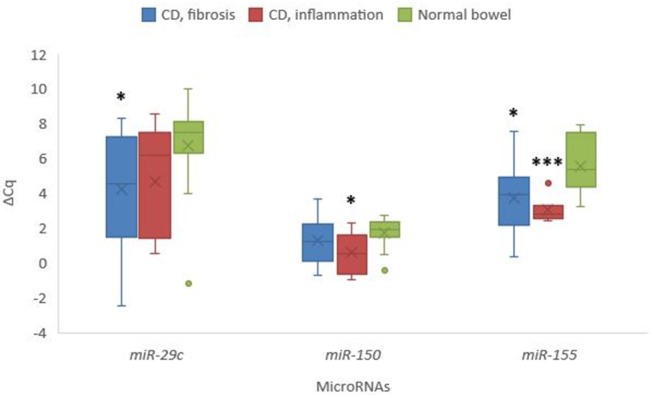
Expression of *miR-29c, miR-150*, and *miR-155* in stenotic and inflamed areas in Crohn's disease in comparison to normal colon mucosa. Legend: CD, Crohn's disease; ^*^p < 0.05; ^***^p ≤ 0.001.

Selected miRNAs expression showed statistically significant deregulation of *miR-29c* and *miR-155* in samples from fibrostenotic areas (*p* = 0.027 and *p* = 0.020, respectively), as well as statistically significant deregulation of *miR-150* and *miR-155* in samples from inflamed areas of CD compared to normal mucosa (*p* = 0.037 and *p* = 0.001, respectively) (Figure 6).

## Discussion

We analyzed the pathologic features of fibrosis in resection specimens of CD, with hypothesis that fibrosis in CD pathogenetically resembles fibrosis in other organs. Masson's trichrome staining demonstrated fibrosis in CD mostly in the submucosa and subserosa, but not in the lamina propria. Comparison between samples from fibrostenotic and inflamed areas in CD showed little differences regarding submucosal fibrosis. Important differences were however observed in the subserosa. In inflamed areas, fibrosis was mostly absent or present only focally, close to the muscularis propria. In samples from fibrostenotic areas, dense fibrosis was seen, extending deep into the subserosal fatty tissue, often reaching the serosal surface. Our first conclusion is that the ≫ hot spots ≪ for fibrosis in CD are the submucosa and subserosa.

Trichrome staining is a simple, widely used method in pathology to illustrate fibrous tissue containing collagens (27). It is generally accepted that collagens are produced by myofibroblasts, the key effector cell in fibrosis in various human diseases (15). Our next step was therefore the analysis of activated myofibroblasts in CD, using immunohistochemistry for α-SMA. Proliferation of α-SMA-positive myofibroblasts was present in CD with stenosis in areas of dense fibrosis in the submucosa and subserosa. In CD with inflammation without fibrosis, α-SMA-positive myofibroblasts were present in the submucosa, particularly close to ulcers. Our second conclusion is that myofibroblasts are present predominantly in areas of fibrosis in the submucosa and subserosa.

The interpretation of α-SMA as an immunohistochemical marker of activated myofibroblasts is demanding in the bowel wall because α-SMA is also expressed by normal structures, such as smooth muscles in the muscularis mucosae and muscularis propria. The situation is complicated further by another feature of CD, that is, smooth muscle hyperplasia and hypertrophy, known to contribute to stenosis (28). Nevertheless, by combining morphology and immunohistochemistry, it is possible to distinguish between myofibroblasts and smooth muscle layers of muscularis mucosae and muscularis propria. Moreover, immunohistochemistry for fibroblast activation protein (FAP) may be helpful as it stains myofibroblasts but not smooth muscles (29), further supporting the described patterns in CD.

Despite a generally accepted central role of myofibroblasts in various physiologic and pathologic conditions, the origin of these important cells has not been entirely established and remains controversial (30). It has been suggested that myofibroblasts originate from multiple sources, including local interstitial fibroblasts, pericytes, endothelial cells, bone-marrow-derived circulating fibrocytes and the injured epithelium itself via epithelial-mesenchymal transition (16, 26, 31–33). However, recent studies on experimental models of kidney fibrosis have provided strong evidence that local mesenchymal cells, which are usually called resident fibroblasts when embedded in the matrix and pericytes when attached to the capillaries, are the major source of myofibroblasts. In response to injury, they detach from the vessels and differentiate into myofibroblasts causing fibrosis, contributing to capillary rarefication and inflammation (18, 34, 35).

We therefore analyzed the distribution of pericytes/fibroblasts in normal bowel and in CD. There is no ideal marker for pericytes and fibroblasts but, on the basis of available literature, we chose CD34, which has proven useful in many previous studies (36). Since CD34 also stains endothelial cells, it is not easy to distinguish between endothelial cells and fibroblasts. We therefore used double immunohistochemistry. With the help of CD34 and erg as markers of endothelial cells (with the cytoplasmic reaction of the former and nuclear reaction of the latter), it was feasible to distinguish the double labeled (CD34+ erg+) endothelial cells with cytoplasmic and nuclear staining from fibroblasts (CD34+ erg-) with cytoplasmic staining only. We found that in normal bowel, pericytes/fibroblasts were abundant in the submucosa and subserosa but not in the lamina propria. They were particularly numerous around blood vessels, and these cells have been referred to as pericytes. It has been suggested recently that some of these cells are in fact mesenchymal stem cells (37).

In CD, a consistent pattern was observed in fibrosis: fibroblasts/pericytes disappeared and myofibroblasts appeared in areas of fibrosis. Milia et al. (38) found a similar distribution of CD34-positive cells referred to as telocytes which also disappeared in CD. This pattern suggests that fibroblasts/pericytes are the most likely origin of myofibroblasts in CD. The extent to which other sources contribute to the pool of myofibroblasts, such as epithelial, endothelial and mesothelial cells via epithelial/endothelial/mesothelial-mesenchymal transition, remains to be determined.

The next conclusion of this study is that the distribution of fibroblasts/pericytes in normal colon and their disappearance in fibrosis, in parallel with the appearance of myofibroblasts, suggests that pericytes/fibroblasts are the source of myofibroblasts in CD.

One of the most important questions is which factors trigger fibrosis in CD. The key event in the pathogenesis of CD is the production of inflammatory cytokines (39). Among them, TGF-β1 is believed to be the most potent profibrogenic cytokine, as demonstrated in various experimental models and in human diseases (40). It induces mesenchymal cell activation and differentiation into myofibroblasts (41, 42). We therefore analyzed TGF-β1 using mRNA *in situ* hybridization and observed positive reaction in mononuclear cells in CD. They were particularly abundant in lymphoid agreggates in areas of deep fibrosis in CD, whereas TGF-β1 was not expressed in deeper layers of the normal intestinal wall. The expression patterns of TGF-β1, the most potent profibrogenic cytokine, thus corresponded well to the distribution of inflammation and fibrosis in CD. Similar expression patterns of TGF-β1 were described by Lawrance et al. (43) stating that expression depended on the presence and location of inflammatory infiltrates.

We also analyzed the expression of three microRNAs which have been demonstrated to be involved in inflammation and fibrosis in various organs (44), and found deregulation of *miR-29c* and *miR-155* in samples from fibrostenotic areas of CD, while deregulation of *miR-150* and *miR-155* was found in samples from inflamed areas of CD compared to normal mucosa. Similar expression patterns were described in fibrosis in other organs (45–47). *miR-29* family is widely accepted as one of the major contributor to fibrosis (45), whereas *miR-150* is recognized as an immune-microRNA, being differentially expressed in inflammation (46), and *miR-155* is a multifactorial microRNA showing differential expression in both fibrosis and inflammation (47). Some of these microRNAs have already been analyzed in CD showing similar results, e.g., *miR-29* family (48) and *miR-155* (48, 49). For all three investigated microRNAs, we observed different levels of expression between inflamed and fibrotic areas, although the differences were not significant. Increasing number of publications suggest that most microRNAs, including *miR-29c, miR-150* and *miR-155* contribute to a wide spectrum of disease processes. Moreover, numerous publications showed that panels of microRNAs and not only one microRNA are needed to detect appropriate signature of the disease or pathogenetic process (45–49). Additional studies are warranted to increase our knowledge about the involvement of microRNAs in CD.

Finally, the distribution of fibrosis, with submucosa and subserosa as hot spots, clearly shows why endoscopic biopsies are not efficient for predicting fibrosis in CD. The most important issue in the future is to search for markers that enable the prediction of fibrosis from endoscopic biopsies early in the course of the disease and identification of patients who are at risk of developing severe fibrosis and stenosis.

Our study has several limitations. As only patients who had undergone surgery were included in the study, a selection bias, with only the most severe cases of CD being analyzed, cannot be excluded. It was also not possible to identify and/or exclude the effect of treatment on the morphological findings. Finally, this study is mostly observational, and immunohistochemistry only enabled analysis of protein expression, without providing any functional proof.

## Conclusion

Fibrosis in CD is similar to fibrosis in other organs, with myofibroblasts as the key effector cell. Pericytes/fibroblasts appear to be the source of myofibroblasts in the bowel wall, as has likewise been shown for other organs. The specificities of fibrosis in CD are related to the structure of the bowel wall. The four layers of the bowel wall, with abundant pericytes/fibroblasts in the submucosa and subserosa, could regulate the response of the bowel wall to injury. When the injury within the bowel wall goes deep (probably beyond the muscularis propria), an extensive scar formation may protect from perforation (50). Fibrosis and stenosis in CD may therefore be regarded as the result of a normal protective (over)response that is prompted by cytokines present within deep structures of the bowel wall.

## Data Availability Statement

The datasets generated for this study are available on request to the corresponding author.

## Ethics Statement

This manuscript is part of a study which was approved by the State Ethical Committee of the Republic of Slovenia (No. 0120-595/2017/5) on 6th Dec, 2017. As stated in the approval document, the study is retrospective, observational, performed on tissue samples that were obtained during routine diagnostic/therapeutic procedures, consisted of either excision or resection. Therefore, enough tissue was available for routine analysis and research. Moreover, tissue is still available for any additional routine, therapy-related or research analyses in the future. Our State Ethical Committee does not require informed consent from patients in such studies. However, the written informed consent was obtained before the routine procedure.

## Author Contributions

NZ and CL contributed conception and design of the study. MJ, DD, and AT organized the database. MJ performed the statistical analysis. NZ wrote the first draft of the manuscript. CL and EB wrote sections of the manuscript. All authors contributed to manuscript revision, read, approved the submitted version, and made substantial contributions to the submitted work.

## Conflict of Interest

The authors declare that the research was conducted in the absence of any commercial or financial relationships that could be construed as a potential conflict of interest.
